# Icariside II Preparation from Icariin Separated from *Epimedium* Herbal Extract Powder by a Special Icariin Glycosidase

**DOI:** 10.4014/jmb.2408.08046

**Published:** 2024-10-24

**Authors:** Xinyu Liu, Siyu Xu, Chunying Liu, Zhenghao Wang, Bo Wu, Meijuan Guo, Changkai Sun, Hongshan Yu

**Affiliations:** 1 College of Biotechnology, Dalian Polytechnic University, Qinggong-Yuan No. 1, Ganjingzi-qu, Dalian 116034, P.R. China; 2 School of Life Science and Biotechnology, Liaoning Marine Microbial Engineering and Technology Center, Dalian University, Xuefu-Dajie No. 10, Economic Technological Development Zone, Dalian 116622, P.R. China; 3 Research & Educational Center for the Control Engineering of Translational Precision Medicine, Dalian University of Technology, Linggong-ru No. 2, Ganjingzi-qu, Dalian 116024, P.R. China

**Keywords:** Icariside II, special icariin glycosidase, powdered *Epimedium* extract, by-product icaritin, bioconversion dynamics

## Abstract

In this study, icariside II was prepared from icariin by a special enzyme. The yield of the substrate icariin from a powdered extract of the popular herb *Epimedium* was 16.9%. The enzyme, which was produced from *Aspergillus* sp.y48 fermentation, hydrolyzes icariin to icariside II and was characterized. The molecular weight was 75 kDa, while the optimum temperature and pH were 45°C and 5.0. The purified enzyme hydrolyzed the 7-*O*-glucoside of icariin or epimedin A, B, and C to icariside II, or sagittatoside A, B, and C, respectively, and further hydrolyzed the terminal 3-*O*-xyloside of sagittatoside B to icariside II. The enzyme is a special icariin glycosidase that hydrolyzed icariin to icariside II at low cost. Based on the crude enzyme’s reaction dynamics, the optimal conditions for icariside II preparation showed that 2% icariin reacted at 45°C for 6 to 9 h. Here, we obtained 13.3 g icariside II and 0.45 g of the by-product icaritin from 20 g icariin. The icariside II molar yield was 87.4%, the by-product icaritin yield was 4.1%, and the total molar yield was 91.5%. Therefore, icariside II was resoundingly prepared from an icariin glycosidase of an *Epimedium* extract using a non-GMO, crude enzyme from *Aspergillus* sp.y48. The obtained icariside II and the by-product icaritin can be directly applied in the production of cosmetics and pharmaceuticals.

## Introduction

Epimedii folium (*Epimedium*, or yinyanghuo in Chinese), the dried stems and leaves of *Epimedium* plants, have good pharmacological activities that can help strengthen muscles and bones, invigorate the kidneys and heart, and treat the weak flow of energy, or “wind dampness.” *Epimedium* species have been used for over 2,000 years in China and Asian countries as a celebrated medicinal herbal medicine [1, 2]. There are about 70 species in the genus *Epimedium* [2], but only four of these (*Epimedium sagittatum*, *Epimedium brevicornum*, *Epimedium koreanum* Nakai, and *Epimedium pubescens* Maxim) are included in the 2020 Chinese Pharmacopoeia [3]. The main active ingredients of *Epimedium* are the flavonoids and the content is over 5% (w/w). About 70 kinds of *Epimedium* flavonoids have been found and about 80 to 85% (w/w) of these ingredients are four type 8-isopentene flavonoids, namely icariin, epimedin A, B and C, while the others were low in content [1, 4]. The structures of the main and rare 8-isopentene flavonoids of *Epimedium* herb are as shown in Fig. S1.

Epimedin A, B and C have three glycosides, and the icariin has two glycosides. In addition, flavonoids such as these with low activities cannot be directly absorbed by the human body. After oral administration, the glycosides of epimedin A, B, C and icariin are hydrolyzed by intestinal enzymes and bacteria into low-glycoside flavonoids, such as icariside II or icaritin, to absorb and exhibit pharmacological efficacy. However, this bioconversion of *Epimedium* flavonoids in the gastrointestinal tract is poor [5-8].

The icariside II with one 3-*O*-rhamnoside is easily absorbed by the body and exhibits useful pharmacological activities [9], including anti-carcinogenic [10, 11] bone-strengthening [12], anti-Alzheimer [13], anti-diabetic [14, 15], and aphrodisiac properties [16], all of which are useful in the production of pharmaceuticals, health foods, and cosmetics. The icaritin also exhibits useful pharmacological activities [17-19] that help in preventing cancer, inflammation, angiocardiopathy and impotence, osteoporosis [20], and Alzheimer’s disease [21].

However, the icariside II content in *Epimedium* is very low and difficult to separate from the plant itself. Thus, in vitro conversions of the major, low-activity *Epimedium* flavonoids into high-activity icariside II are very useful in developing drugs from *Epimedium*.

The biotransformation of *Epimedium* flavonoids has been widely studied in the effort to obtain rare *Epimedium* flavonoids such as icariside II. Yang *et al*. purified a special icariin glycosidase from a fungal strain with a molecular weight of 65 kDa; however, the enzyme icariside II production was very low [22]. Using a crude snailase, Gao *et al*. hydrolyzed the glycosides of epimedin A, B, and C, and icariin, to sagittatoside A, B, and C, and icariside II, and further to icaritin, but again the icariside II production was poor [23]. Li *et al*. converted icariin into icaritin with β-glycosidase; however, its reaction efficiency was low [24]. Zhang *et al*. hydrolyzed a mixture of epimedin A, B, C and icariin using the thermostable recombinant enzymes of β-glucosidase and α-L-rhamnosidase to prepare icaritin, but the product was not icariside II, and the icaritin weight yield was only 4% [25]. In addition, Wang *et al*. hydrolyzed icariin to prepare icaritin by a special *Epimedium* flavonoid glycosidase (molecular weight, 73.2 kDa) from *Aspergillus* sp.y848 strain, but there was no icariside II production [26].

The icariside II in this study was prepared from icariin by a special icariin glycosidase. The substrate icariin was separated from a popular *Epimedium* extract powder containing over 20% (w/w) icariin. The special icariin glycosidase that hydrolyzed icariin to icariside II was prepared and characterized from the fermentation of *Aspergillus* sp.y48 strain. Moreover, the crude enzyme was used in the preparation of icariside II from icariin at low cost. Although the crude enzyme contained a trace of the enzyme hydrolyzing icariside II into a small amount of icaritin, it had no effect on the icariside II preparation. The enzyme reaction and dynamics of icariin were studied to ascertain the optimal conditions of icariside II preparation from icariin by the crude enzyme. The icariside II was produced from icariin by crude enzyme, and a small amount of by-product icaritin was also obtained. The structures of the icariside II and by-product icaritin were determined using NMR spectroscopy.

## Materials and Methods

*Aspergillus* sp.y48 strain was previously isolated from daqu (Chinese traditional fermenting agent) [26-29]. Flavonoid standards such as icaritin, icariside II, epimedin A, epimedin B, epimedin C, icariin, sagittatoside A, sagittatoside B, and sagittatoside C were products of Guangrun Biotech Co. Ltd. (China). The powdered extract of *Epimedium*, containing over 20% icaritin, was obtained from Xi'an Yueda Plant Technology Co. Ltd. (China). Macroporous ion exchange resin D-280 was a product of Tianjin Yunkai Resin Technology Co. Ltd. (China). Silica gel plate 60-F254 was purchased from Merck (Germany). The marker proteins included phosphatase b (97.4 kDa), bovine serum albumin (66.2 kDa), actin (43 kDa), carbonic anhydrase (31 kDa), trypsin inhibitor (22 kDa), and lysozyme (14.4 kDa) and were products of TaKaRa Bio Inc. (China).

### Icariin Isolation from Powdered Extract of *Epimedium*

To obtain the substrate icariin, the powdered extract of *Epimedium* was dissolved in 10× (w/v) 45% ethanol (v/v), eluted on the same volume column of macroporous ion exchange resin D-280 to absorb the *Epimedium* flavonoids and decolorize. The flavonoids on the column were eluted by 50% ethanol (v/v) (12-15× volume of column) until there was no icariin by TLC. The ethanol solution was concentrated to precipitated icariin, which was collected, washed twice with cold 45% ethanol (v/v), and dried to obtain the substrate icariin. Icariin was then used in the subsequent enzyme conversion experiments.

### Enzyme Production and Purification

To select a strain that efficiently produces the special enzyme hydrolyzing icariin to icariside II, the *Aspergillus niger* g.848 strain [28], *Aspergillus niger* b.48 [29], and *Aspergillus* sp.y48 were cultured in 200 ml of medium containing 0.4% *Epimedium* extract powder and 5% malt extract in a 1,000 ml conical flask, with 50-60 rpm shaking at 28-30°C for 5 to 6 days. The results showed that the *Aspergillus* sp.y48 strain efficiently produced the special enzyme hydrolyzing icariin to icariside II. The cell growth, enzyme activity, and substrate-maltose reduction in the fermentation of *Aspergillus* sp.y48 strain were determined by the method of Jin et. al. [30] and Wang et. al [31] to ascertain the optimal culture conditions of enzyme production.

The crude enzyme from the *Aspergillus* sp.y48 strain was purified based on a column fraction method using DEAE cellulose (DE-52, Whatman Inc., England) [27, 29]. Briefly, the crude enzyme solution was added to the DEAE cellulose DE-52 ion exchange column, which was then washed with 20 mmol/l acetic acid buffer (pH 5.0), followed by elution with different concentrations of KCl solution. The collected enzyme solution was reacted with icariin respectively, and the conversion of icariin to icariside II was confirmed by TLC. The enzyme molecular weight was determined by SDS-PAGE [32]. The concentration of protein was measured by Folin phenol reagent using bovine albumin as standard protein [32, 33].

One unit of special icariin glycosidase activity was the enzyme amount reducing 1 M icariin/min at 45°C.

### Enzyme Properties

The optimal pH and temperature of the purified icariin glycosidase from the *Aspergillus* sp.y48 strain were determined in 0.2% icariin (w/v) at pH 4.0, 4.5, 5.0, 5.5, 6.0 and 7.0 at temperatures of 35, 40, 45, 50, 55 and 60°C with reaction times of 2-4 h by the purified enzyme, respectively.

To determine the hydrolysis abilities of the purified enzyme on glycosides of epimedin A, B, C, and icariin, 0.1 ml purified enzyme was mixed with 0.1 ml of 0.4% different substrates in 0.02 M and pH 5.0 acetate buffer (final substrate, 0.2%) to react at 45°C for 2-7 h, respectively. Then, 0.2 ml water-saturated *n*-butanol was added to the enzyme reaction solution. The products and substrate in the *n*-butanol layer were measured using the HPLC and TLC methods, respectively.

To obtain the purified enzyme kinetic constants, 50 L purified enzyme was mixed with 50 L of substrate solutions of icariin with a series of concentrations of 0.8, 1.0, 1.34, 2.0, and 4.0 mmol/l (final substrate concentrations were 0.4, 0.5, 0.67, 1.0, and 2.0 mmol/L) to react at 45°C for 9 h. Then, 100 L of water-saturated *n*-butanol was added to terminate the reaction. The products and substrate were measured using the HPLC and TLC methods, respectively. The enzyme kinetic constants *V*_m_, *K*_m_, *K*_cat_, and *K*_cat_/*K*_m_ were calculated using the Lineweaver Burk plotting method [34].

To ascertain the specificity of the purified enzyme, the enzyme activity was studied on different type of substrates as shown in Table S1. The substrate concentrations were all 2.0 mg/ml. After standing at 45°C for 10 min, 0.1 ml of purified enzyme solution was added to 0.15 ml of substrate solution to react under optimal reaction conditions for 30 min. Then, 2.5 ml of 1 mol/l Na_2_CO_3_ solution was added and distilled water was used as the blank group.

### Dynamics of Icariin Reaction by Crude Enzyme

To obtain the optimum pH and temperature of the crude enzyme, 4% icariin was mixed with the same volume of crude enzyme, and reacted for 6 h in pH 4.0, 4.5, 5.0, 5.5, 6.0 and 7.0, at temperatures of 35, 40, 45, 50, 55 and 60°C, respectively.

To obtain the optimal icariin concentration, 2, 3, 4 and 5% (w/v) icariin in 0.02 M and pH 5.0 acetate buffer were mixed with the same volume of crude enzyme (final icariin concentrations were 1.0, 1.5, 2.0 and 2.5%), and reacted at 45°C and pH 5.0 for 6 to 9 h, respectively.

To determine the optimal enzyme reaction time, the dynamics of crude enzyme hydrolysis on icariin were analyzed. Using the crude enzyme, 2.0% icariin was reacted at 45°C for 0.5 to 28 h.

In the determination of the icariin conversions at different times, 0.1 ml of reacted solutions was mixed with 0.2 ml of water-saturated *n*-butanol, and the products and substrate in the *n*-butanol layer were analyzed by the HPLC and TLC methods, respectively.

### Icariside II Preparation from Icariin by Crude Enzyme

The aforementioned icariin was completely mixed in 25× 0.02 M and pH 5.0 acetate buffer to get 4% icariin (w/v). Then, 4% icariin was mixed with the same volume of crude enzyme (final icariin, 2%) in a 10 L bioreactor (Henan Taihongsheng Equipment Co. Ltd., China), and reacted at 45°C for 6 to 12 h with stirring at 65 rpm. After the icariin was completely reacted by TLC, the product precipitates were collected by centrifugation. The precipitates were washed 3-4 times with water, and then dried to obtain crude products. The dried, crude products were dissolved in 12-15× tetrahydrofuran (w/v), and then filtered to remove impurities. The filtered solution was mixed with 4× volume 50% methanol (v/v) of tetrahydrofuran to precipitate the by-product icaritin, which was then filtered and purified using the reference method [26]. The supernatant containing icariside II was vacuum-concentrated and dried to obtain the icariside II product.

### TLC, HPLC and NMR Analyses

The *Epimedium* flavonoids were analyzed using TLC plates of 60-F254 silica with the solvent consisting of ethyl acetate: butanone: water: methanol = 8: 7: 1: 1 (v/v/v/v). The plate spots of TLC were colored at 280 nm ultraviolet. The content ratio of products and substrate was analyzed using Bandscan software based on the plot area and plot shade of the plate [27].

In the HPLC determination of enzymatic flavonoid products and substrates, the equipment used was a Waters 2695 Separations Module (Waters, USA) with the Waters 2996 Photodiode Array Detector, using the Zhonghuida C18 column (5 μm, 4.6 mm × 250 mm). The mobile phase was A (acetonitrile) and B (water): from 0 to 8 min, A 17 to 27% (v/v); from 8 to 32 min, A was 27% (v/v); from 32 to 60 min, A from 27 to 85% (v/v); from 60 to 70 min, A from 70 to 80% (v/v); and from 70 to 80 min, A from 85 to 100% (v/v). The wavelength was 273 nm. The injected volume was 10 L with a flow rate of 1 ml/min, and the column temperature was 35°C.

In the HPLC determination of protein, the column was a TSKgel G2000SWxl (7.8 mm × 300 mm, Tosoh Bioscience, Japan) [27, 28]. The mobile phase was 0.05% (w/v) sodium azide in 0.02 M and pH 6.7 phosphate buffer; the wavelength was 280 nm; the flow rate was 1.0 ml/min; and the injected volume was 100 L [27, 28].

In the NMR analysis, the icariside II product and by-product icaritin were dissolved in DMSO-*d6* or Pyridine-*d5*, and the spectra (^13^C, 150 MHz; ^1^H, 600 MHz) were determined using Bruker’s Avance II Spectrometer (Switzerland).

## Results and Discussion

### Separation of Icariin from *Epimedium* Extract Powder

**Fig. 1:**
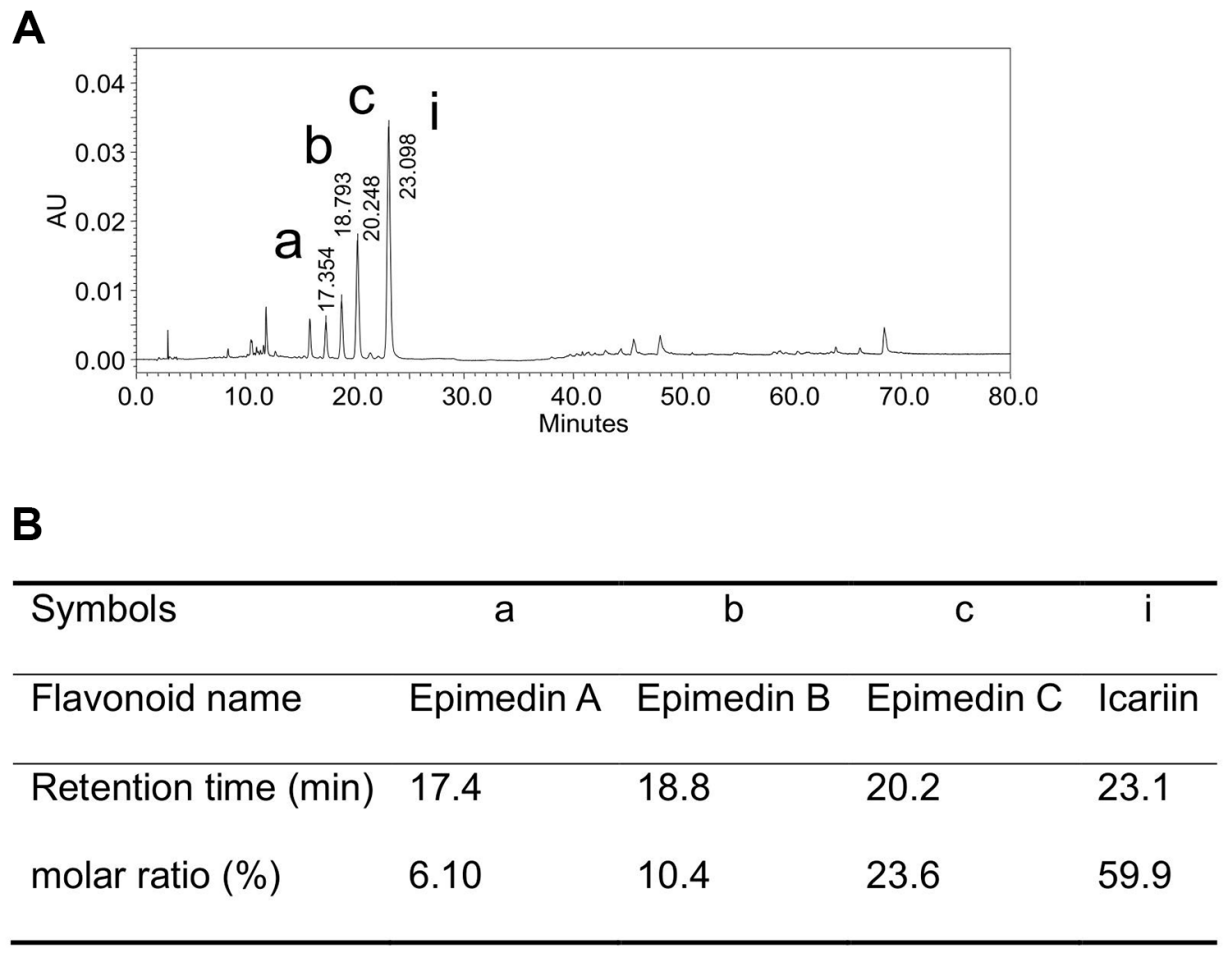
Flavonoid consists of *Epimedium* herb extract powder. (**A**) Flavonoids of of *Epimedium* herb extract powder in HPLC. (**B**) Table of main flavonoid molar ratio (%) from HPLC.

To obtain the icariin substrate, the flavonoid content of a popular *Epimedium* herbal extract powder was first determined by the HPLC method. The total flavonoid content was about 40%. If the HPLC peak area ratio of *Epimedium* flavonoids was assumed to be the flavonoid molar content ratio, or the flavonoid content ratio, then the content ratio of the main *Epimedium* flavonoids was 23.5% epimedin C, 10.4% epimedin B, 6.02% epimedin A, and 59.9% icariin (Fig. 1).

In the icariin separation, 150 g of powdered extract of *Epimedium* was dissolved in 1,500 ml 45% ethanol (v/v), and eluted on the 1,500 ml column of macroporous ion exchange resin D-280 to adsorb the *Epimedium* flavonoids, and decolorize. The flavonoids on the column were gradually eluted by the 50% ethanol (v/v) (12-15× volume of column) until there was no icariin. The ethanol solution was combined and concentrated to precipitated icariin, which was then collected using a filtering method, washed three times with a small amount of cold 45 or 50% ethanol (v/v), dried to get 25.3 ± 1.2 g icariin (average of three experiments) from 150 g of *Epimedium* extract powder. The experiment was repeated 3 times. The weight yield of icariin was 16.9% and the purity was over 90% by HPLC. The prepared icariin was used in subsequent experiments.

### Enzyme Production

To select a strain that efficiently produces the special enzyme hydrolyzing icariin to icariside II, the strains *Aspergillus niger* g.848 [28], *Aspergillus niger* b.48 [29], and *Aspergillus* sp.y48 were cultured at 28-30°C for 5-6 days, with 50-60 rpm shaking of 200 ml medium in a 1,000 ml conical flask, respectively. The medium contained 5% malt extract and 0.4% *Epimedium* extract powder. The highest enzyme production was obtained from the culture of *Aspergillus* sp.y48 strain.

To obtain the optimum concentration of malt extract and *Epimedium* extract powder (enzyme inducer) for the production of icariside II from icariin using a special icariin glycosidase, the *Aspergillus* sp.y48 strain was cultured in medium containing 4, 5, or 6% (w/v) malt extract, and 0.3, 0.4, or 0.5% (w/v) *Epimedium* powder (as enzyme inducer) at 28-30°C for 6 days with 50-60 rpm shaking, respectively. The optimum concentration of malt extract was 5% (w/v) and the optimum enzyme inducer concentration of *Epimedium* extract powder was 0.4% (w/v).

**Fig. 2:**
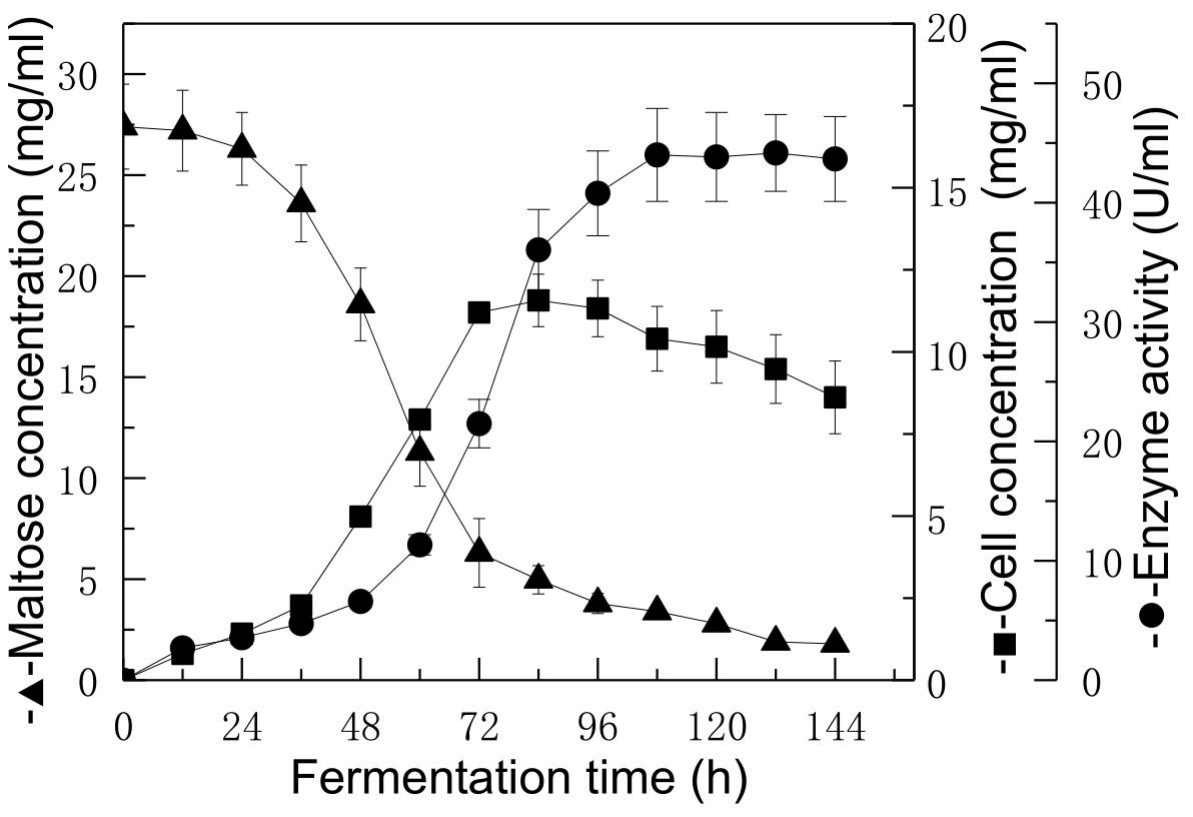
Enzyme production behavior by the cultutre of *Aspegillus* sp. y48 strain. *Aspergillus* sp.y 48 strain was cultured in the medium of 5% (w/v) mal extract and 0.4% (w/v) *Epimedium* herb powder at 28-30°C. ▲, substrate moltose (mg/ml); ■, cell growth (mg/ml); ●, enzyme production (U/ml).

To determine the optimal time of enzyme production in the culture of *Aspergillus* sp.y48 strain, the behaviors for the cell growth, substrate maltose reduction and enzyme production were all determined by reference methods [33] in the medium of 5% (w/v) malt extract and 0.4% (w/v) *Epimedium* extract powder (Fig. 2).

As shown in Fig. 2, during the culture, the cell concentration increased during 72 h, and the maximum was achieved in the 84 h culture, and then it slightly decreased. The substrate maltose decreased rapidly in the 72 h culture. The enzyme production was increased to maximum after culture for 108-132 h (5-6 days); the fermentation was longer than the 48 h fermentation time of common sugar glycosidase or protease by Aspergillus strain [33, 35]. Thus, the fermentation of the special enzyme hydrolyzing icariin to icariside II by *Aspergillus* sp. y48 strain was a long fermentation, and an enzyme inducer was also needed. These results were similar to the long fermentation of other natural-product enzymes, such as dioscin glycosidase [33], ginsenosidase [35], and the enzyme hydrolyzing icariin to icaritin [26].

Therefore, the special icariin glycosidase hydrolyzing icariin to icariside II was cultured at 28-30°C for 108-132 h (5-6 days) by the *Aspergillus* sp.y48 strain in the medium of 5% (w/v) malt extract and 0.4% (w/v) *Epimedium* extract powder. Here, 2 L of culture from ten 1,000 ml conical flasks by *Aspergillus* sp.y48 strain was freeze-centrifuged to remove the cells, then the supernatant was mixed with 3× methanol to precipitate the enzyme protein which was then stored overnight. The precipitated enzyme protein was collected using freeze-centrifugation and dissolved in 200 ml of 0.02 M and pH 5.0 acetate buffer. Non-dissolved impurities were then removed by freeze-centrifugation to obtain the crude enzyme. The experiment was repeated 5 times.

### Enzyme Purification and Characterization

The crude enzyme hydrolyzing icariin to icariside II from the fermentation of *Aspergillus* sp.y48 strain was purified by the method of Wang *et al*. [26] and Yu *et al*. [29]. Ten milliliters of crude enzyme was eluted on a DEAE-Cellulose DE-52 column to adsorb enzyme protein. Then, the enzyme protein of the column was eluted and fractioned by the stepwise method with 100 ml of 0.01 M and pH 5.0 acetate buffer containing 0.06, 0.12, 0.18, 0.24, 0.30, 0.36, and 0.42 M KCl (with fractions of 3.0 ml/tube), respectively. In the enzyme activity determination of the fractions, 0.1 ml of the enzymes was mixed with 0.1 ml of 0.4% icariin (w/v) in 0.01 M and pH 5.0 acetic buffer (final icariin, 0.2%), and reacted at 45°C for 2-6 h. Then, 0.2 ml water-saturated *n*-butanol was added, and the products and substrates in the butanol layer were analyzed by the HPLC and TLC methods, respectively. The enzyme protein purities of the fractions were analyzed by the methods of protein HPLC and SDS-PAGE [31].

**Fig. 3:**
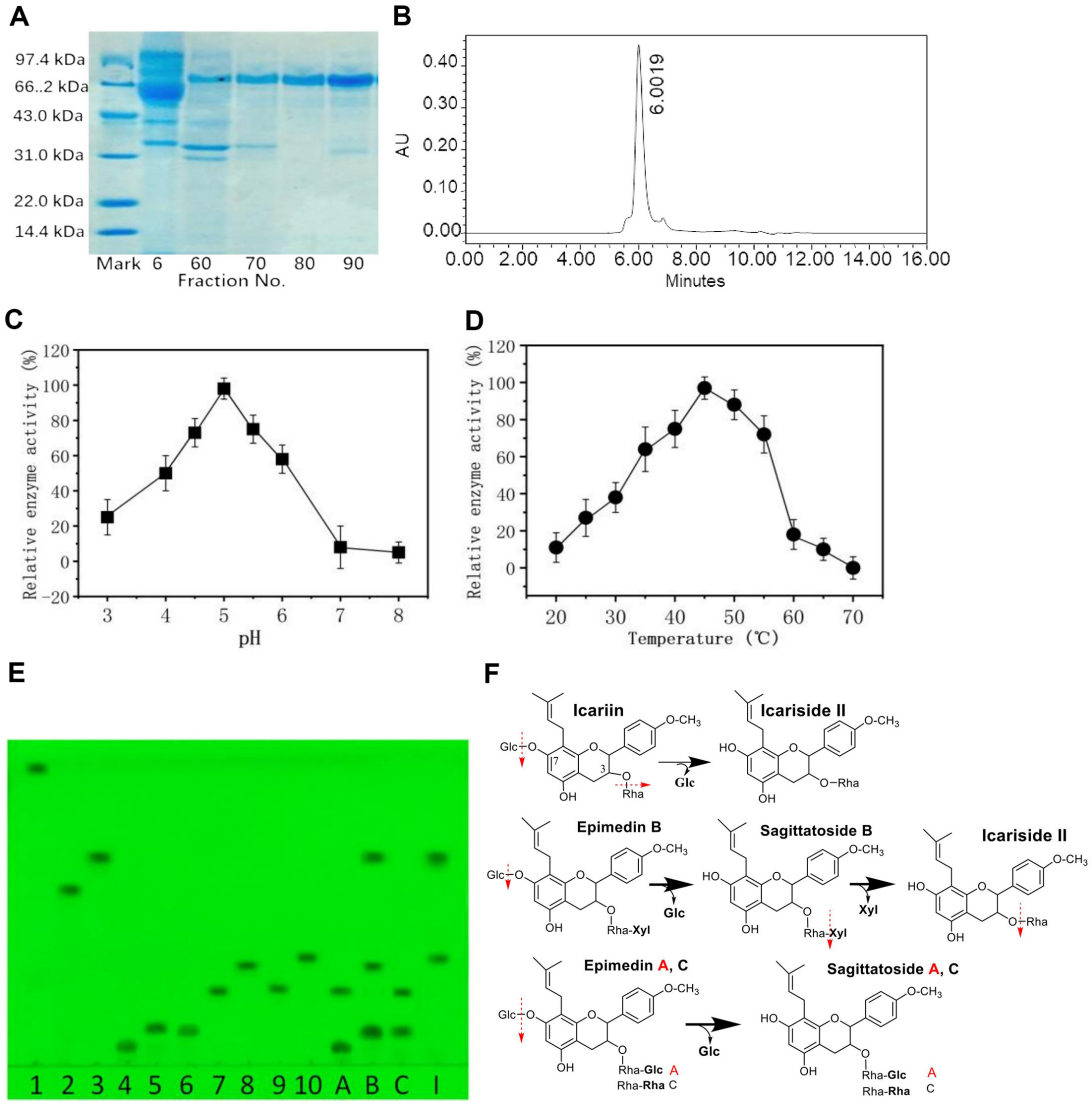
Purified special icariin-glycosidase in SDS-PAGE, enzyme optimal pH and temperature, and hydrolysis on the icariin, epimedin A, B and C. (**A**) Purified special icariin-glycosidase in SDS-PAGE; Mark, standard proteins: phosphatase b (97.4 kDa), bovine serum albumin (66.2 kDa), actin (43 kDa), carbonic anhydrase (31 kDa), trypsin inhibitor (22 kDa) and lysozyme (14.4 kDa); 6, 60, 70, 80 and 90: the proptein bands of fractions 6, 60, 70, 80 and 90, respectively from DEAE-Cellulose DE-52 column. (**B**) Protein HPLC of the mixture of fractions 78 to 83. (**C**) Optimal pH of purified enzyme, 0.2% icariin was reacted at 45°C for 1-2 h. (**D**) optimal temperature of purified enzyme, 0.2% icariin was reacted at pH 5.0 for 1-2 h. (**E**) Purified enzyme hydrolysis on icariin, epimedin A, B and C in TLC of 60-F254 silica gel plate. 1 to 10, standard flavonoids; 1, icaritin; 2, icariside I; 3, icariside II; 4, epimedin A; 5, epimedin B; 6, epimedin C; 7, sagittatoside A; 8, agittatoside B; 9, agittatoside C; 10, icariin. A, reacted epimedin A; B, reacted epimedin B; C, reacted epimedin C; I, reacted icariin. Developing solvent, ethyl acetate: butanone: water: methanol = 8: 7: 1: 1 (v/v/v/v), the colour of spots were rendered at 280 nm ultraviolet. 0.2% icariin, epimedim A, B and C was reacted at 45°C for 1-2 h, respectively. (**F**) Chemical equations of purified enzyme hydrolysis on icariin, epimedin B, A and C.

The results showed that the enzymes of the fractions 6, 60, 70, 80, and 90 hydrolyzed the icariin to icariside II. However, only the protein of fraction 80 showed a single peak in the protein HPLC (Fig. 3B) and a single band in the SDS-PAGE method; however, others of the fractions 6, 60, 70 and 90 did not show a single band in SDS-PAGE (Fig. 3A), thereby proving that only the enzyme of fraction 80 was a pure enzyme. Further detection of fractions 79, 81, 82 and 83, around fraction 80, showed that all enzymes could hydrolyze icariin to icarisde II, and the enzyme protein was a single band in SDS-PAGE; the mixture of enzymes of fractions 79 to 83 was also a single band in SDS-PAGE (Fig. S2A), and a single peak in the protein HPLC (Fig. S2B), thereby proving that the enzyme of fractions 79 to 83 was a pure enzyme protein.

In careful order, the purity of the purified special icariin glycosidase was further examined by the enzyme band-cutting method using slab-PAGE gel [26, 29]. The results showed that the enzyme protein also had a single peak in the protein HPLC, and a single band in SDS-PAGE. The specific activity of further purified icariin glycosidase was the same as that of the purified enzyme in the procedure using the DEAE-Cellulose column. Therefore, the enzyme hydrolyzing icariin into icariside II was almost purified in the DEAE-Cellulose column procedure.

Based on the protein plot log and mobility of the marker proteins [30, 31] in SDS-PAGE as shown in Figs. 3A and S2A, the enzyme molecular weight of enzyme fraction 80, and the mixture of fractions 79 to 82 was approximately 75 kDa; optimal pH was 5.0 (Fig. 3C), and optimal temperature was 45°C (Fig. 3D). In the enzyme purification, the yield of pure enzyme was 5.7%, and the enzyme specific activity was increased 13.4× fold (Table S2). K^+^, Na^+^, Mg^2+^ and Ca^2+^ ions had little effect on enzyme activity. Fe^3+^ and Cu^2+^ ions inhibited the enzyme activity (Table S3). The relationship between the enzyme reaction rate and the substrate concentration was obtained using the Lineweaver Burk method (Fig. S3) [34]. The *K*_m_, *V*_max_, *K*_cat_ and *K*_cat_/*K*_m_ values were 3.55 mM, 0.89 mM/h, 0.021/s and 0.059 (mmol/l)^-1^ s^-1^, respectively.

The purified enzyme mainly hydrolyzed the 7-*O*-glucoside of epimedin A, B, and C or icariin to the sagittatoside A, B, and C or icariside II, respectively. It also hydrolyzed the terminal 3-*O*-xyloside of sagittatoside B to icariside II; but did not hydrolyze the 3-*O*-glycosides of the sagittatoside A and sagittatoside C as shown in Figs. 3E and 3F. To ascertain this, an assay with different *p-Nitrophenyl* (*p*NP) esters was also performed. The purified enzyme was found to be most active with *p*NP-β-D-glucopyranoside and *p*NP-β-D-cellobioside. The purified enzyme showed lesser activity with *p*NP-α-D-galactopyranoside and *p*NP-β-D-galactopyranoside, and even no activity with other *p*NP esters (Table S1). Thus, the purified enzyme from the *Aspergillus* sp.y48 strain is a special icariin glycosidase hydrolyzing icariin to icariside II.

The purified icariin glycosidase (75 kDa) from *Aspergillus* sp.y48 strain was different from the previously reported icariin enzyme (65 kDa) from Absidia sp.E9r strain [22]. Moreover, it was different from the special icariin glycosidase (73.2 kDa) hydrolyzing icariin to mainly produce icaritin from the *Aspergillus* sp.y848 strain [26].

The special icariin glycosidase from the *Aspergillus* sp.y48 strain was used in the icariside II preparation from icariin, which showed a content of about 60% of the total flavonoids in *Epimedium* [5].

### Determination of Optimal Reaction Conditions for the Crude Enzyme

It was discussed earlier that the purified enzyme yield was only 5.7%, and the process of enzyme purification was troublesome. Thus, the crude enzyme was used for icariside II preparation from the icariin at low cost. However, the crude enzyme from *Aspergillus* sp.y48 strain contained a trace amount of the enzyme hydrolyzing icariside II to icaritin, so the effects of the crude enzyme on the icariside II preparation from icariin were examined in subsequent experiments.

The optimal temperature of crude enzyme from *Aspergillus* sp.y48 strain was 45°C, and optimal pH was 5.0; these were the same as the purified enzyme (Figs. 3C and 3D). To obtain the optimal substrate concentration of enzyme reaction, 2, 3, 4 and 5% icariin mixtures (w/v) were completely mixed in the 0.02 M and pH 5.0 acetate buffer, which was then mixed with same volume of crude enzyme (final 1, 1.5, 2 and 2.5%), and reacted at 45°C for 6 to 9 h, respectively. As a result, the icariin completely reacted to icariside II by TLC in 1, 1.5 and 2% icariin concentration, but the icariin did not react to icariside II in 2.5% icariin concentration. So, the concentration of icariin substrate was defined as 2% (w/v).

**Fig. 4:**
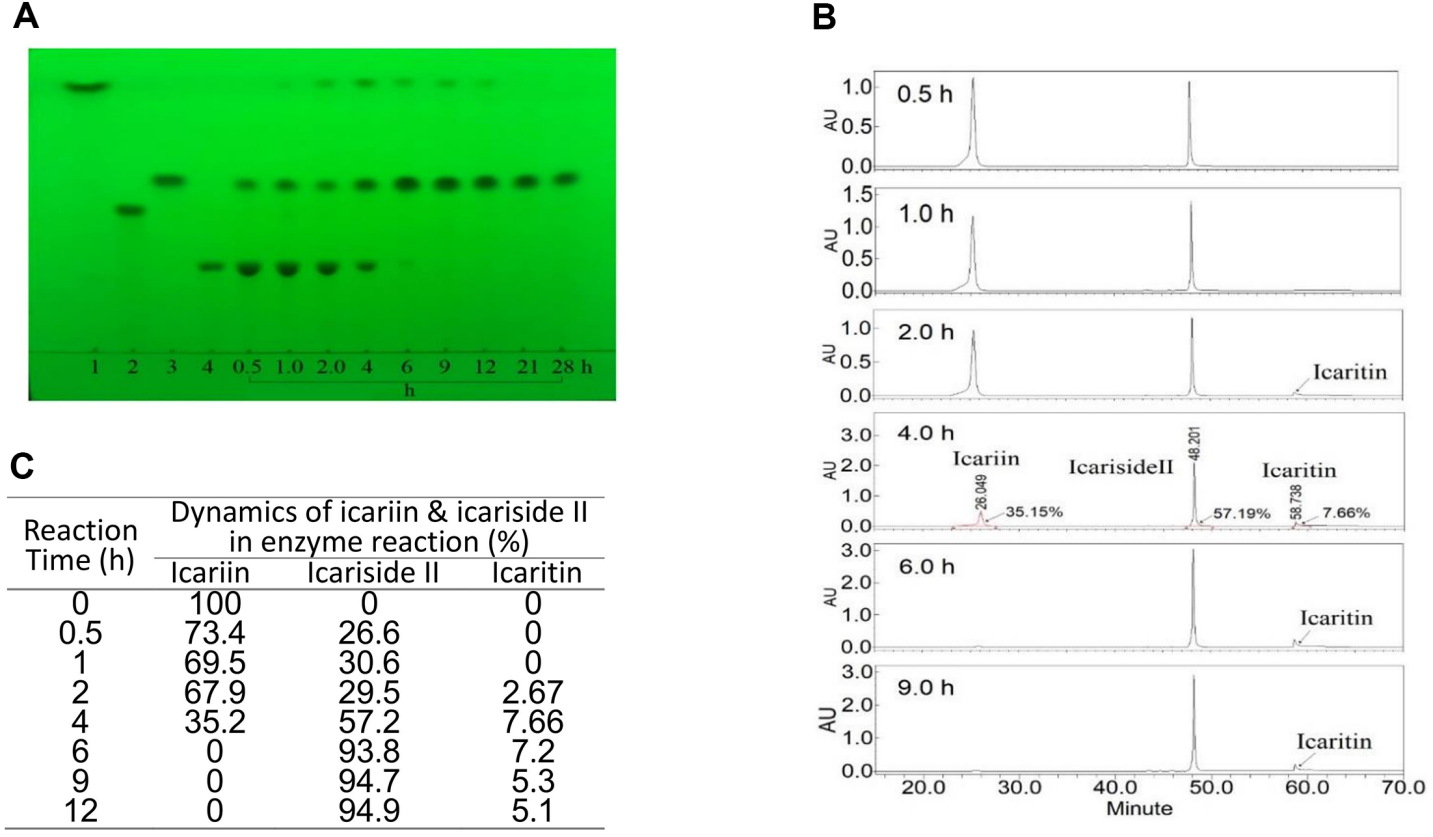
Icariin dynamic changes in different reaction time by the crude enzyme from Aspergillis sp.y 48 strain. (**A**) 2% icariin was reacted at 45°C (pH 5.0) for 0.5, 1, 2.0… to 28 h in TLC. Developing solvent, ethyl acetate: butanone: water methanol = 8: 7: 1: 1 (v/v/v/v); rendering colour in 280 nm ultraviolet. (**B**) Changes of icariin and enzymatic products of icariside II and icaritin by crude enzyme reaction at different reaction time in HPLC. 2% icariin was reacted at 45°C for 0.5, 1.0, 2.0, 4.0, 6.0 and 9.0 h times. (**C**) Table of data of icariin chenges in the different reaction time by crude enzyme from HPLC figures.

To obtain the optimal icariin reaction time by the crude enzyme, the enzyme reaction dynamics were analyzed: 2% (w/v) icariin reacted at 45°C for different times by the crude enzyme with 65 rpm stirring. During the reaction, 0.1 ml solution of different reaction times was mixed with 0.2 ml water-saturated *n*-butanol, and the products and substrate in the *n*-butanol layer were analyzed by the HPLC and TLC methods, respectively (Fig. 4).

We observed from the TLC of Fig. 4A that 2% icariin was reacted for 6 h and the icariin completely reacted to produce the main product icariside II and a small amount of icaritin by-product. When the reaction time was extended from 9 h to 28 h, the product composition of icariside II and the icaritin by-product were mostly unchanged (Fig. 4A).

If the peak area ratio of flavonoids by HPLC was assumed to be the flavonoid content ratio (Fig. 4B), when 2%icariin was reacted for 0.5 h by crude enzyme, the 26.6% icariin was converted to icariside II; when reacted for 4 h, the 30.5% icariin was changed to 57.2% icariside II and 7.66% icaritin by-product. When reacted for 6 h, the icariin was completely converted to 93.8% icariside II and 7.2% icaritin by-product. When reacted for 9 h, the icariin was completely converted to 94.7% icariside II and 5.3% by-product icaritin. After an extended reaction time, the product composition of icariside II and icaritin by-product barely changed (Fig. 4A and 4B).

As shown earlier, although the crude enzyme contained a trace of enzyme hydrolyzing icariside II into icaritin, when reacted for over 9 h, 94.7% of the product was icariside II, and only 5.3% was icaritin. Therefore, the crude enzyme did not have any effect on the icariside II preparation. Hence, the enzyme optimal reaction conditions for icariside II preparation from icariin were defined such that 2% icariin reacted at 45°C for 6-9 h by the crude enzyme from *Aspergillus* sp.y48 strain.

### Icariside II Preparation from Icariin by the Crude Enzyme

According to above results, the optimal reaction conditions for icariside II preparation from icariin by the crude enzyme from *Aspergillus* sp.y48 strain showed that 2% icariin reacted at 45°C for 6-9 h.

Twenty grams of icariin (from 118 g *Epimedium* extract powder) were mixed with 500 ml 0.02 M (pH = 5.0) acetate buffer, and mixed with 500 ml crude enzyme in a bioreactor, and reacted at 45°C for 6-9 h with stirring at 65 rpm. After the icariin was completely reacted by TLC, the product precipitates were collected by centrifugation. The precipitates were washed 4 times with water and dried to obtain crude products. The crude products were dissolved in 12-15× tetrahydrofuran (w/v), and filtered to remove the impurities. Then a 4× volume of 50%methanol (v/v) was added to the tetrahydrofuran solution to precipitate the by-product icaritin, and stored for over 12 h. The precipitated by-product icaritin was collected by centrifugation, washed with a small amount of cold 45 to 50% methanol, and dried to obtain pure by-product icaritin. The supernatant containing icariside II was dried to obtain the pure icariside II product.

**Fig. 5:**
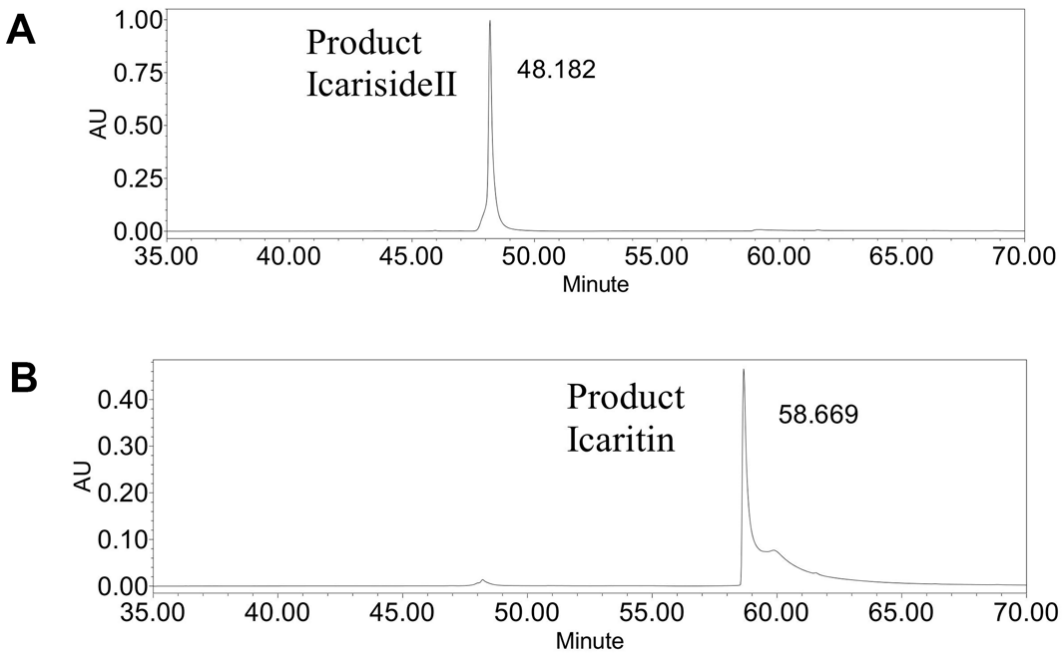
Purity of products icariside II and icaritin by-product from icariin by crude enzyme in HPLC. (**A**) Products icariside II. (**B**) By-product icaritin.

Here, 13.3 ± 1.4 g icariside II (M.W., 514.5, 25.8 mol), and 0.45 ± 0.07 g by-product icaritin (M.W., 368.4, 1.22 mol) were obtained from 20 g icariin (M.W., 676.7, 29.56 mol), that is, from 118 g *Epimedium* extract powder. The main product icariside II weight yield from *Epimedium* extract powder was 11.3%. The icariside II molar yield from icariin was 87.4%; the icaritin by-product molar yield was 4.1%; and total molar yield of products was 91.5%. The icariside II purities were over 90% (Fig. 5A); the icaritin purity was 95% by HPLC (Fig. 5B). These results were the average data of three experiments.

Therefore, the icariside II was resoundingly prepared from icariin isolated from *Epimedium* extract by a non-GMO, crude enzyme from *Aspergillus* sp.y48 strain at low cost, and additionally, a small amount of by-product icaritin was obtained.

### Structure of Icariside II and Icaritin By-Product by NMR

**Fig. 6:**
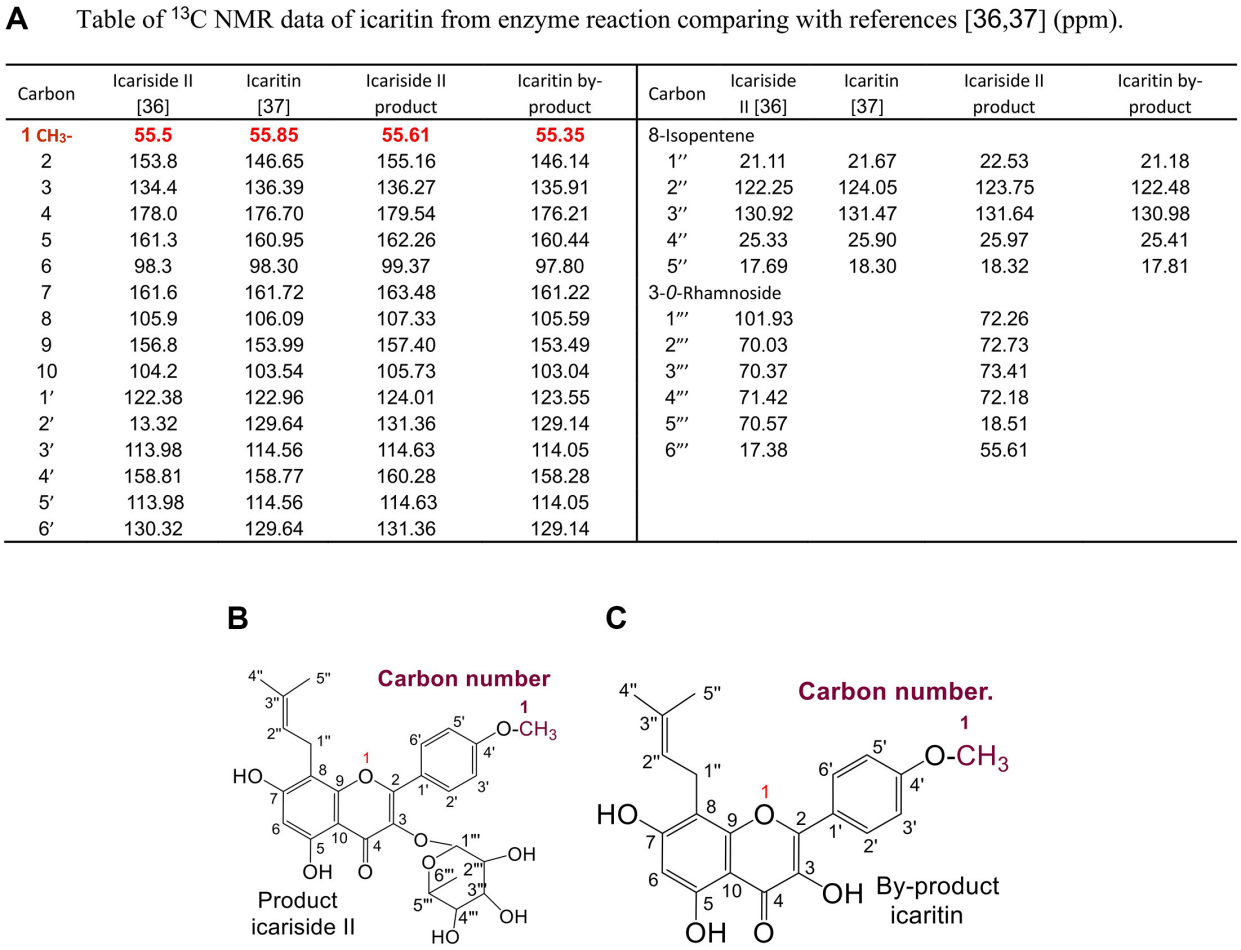
Structure of main product icaruside II and by-product icaritin from enzyme reaction by NMR. (**A**) Table of ^13^C NMR data of products icariside II and icaritin by enzyme reaction comparing with the data of references [36, 37] (ppm). (**B**) Structures of products icaruside II from enzyme reaction. (**C**) Structures of by-product icaritin from enzyme reaction.

The icariside II product and the by-product icariin from the enzyme reaction can be recognized by the HPLC with standard icariside II and icariin. To obtain accurate results, the structures of the icariside II product and the icaritin by-product from the enzyme reaction were determined by NMR. ^13^C NMR (150 MHz) spectral data of icariside II product and by-product icaritin (in DMSO-*d6* or Pyridine-*d5*) are as shown in Figs. 6A and S4, and S5. The structures of icariside II and icaritin were also analyzed by ^1^H, ^13^C, DEPT, HSQC, HMBC, and COSY NMR methods (Figs. S4 and S5). According to previously reported NMR data [35, 36], the main product from enzyme reaction should be icariside II, *i.e.*, 3-*O*-L-Rha-icaritin (Fig. 6B); the by-product should be icaritin, that is, 3,5,7-trihydroxy-2-(4-methoxyphenyl)-8-(3-methyl-2-butenyl)- 4H-1- benzopyran-4-one (Fig. 6C).

## Conclusion

Minor icariside II was prepared from icariin separated from *Epimedium* extract powder by a special enzyme of *Aspergillus* sp.y48 strain. The flavonoid content in the *Epimedium* extract powder was about 40% (w/w), which was composed of 59.9% icariin, 23.5% epimedin C, 10.4% epimedin B, and 6.02% epimedin A. In addition, 25.3 g of substrate icariin was isolated from 150 g of *Epimedium* extract powder; the icariin weight yield was 16.9% and the purity was over 90%.

The special enzyme hydrolyzing icariin to icariside II was produced by the *Aspergillus* sp.y48 strain fermentation in a medium containing 0.4% (w/v) of *Epimedium* extract powder and 5% (w/v) of malt extract. The enzyme was nearly purified from crude enzyme by the DEAE-cellulose DE-52 column. The enzyme molecular weight was about 75 kDa. The enzyme optimal temperature was 45°C and the optimal pH was 5.0. The purified enzyme can hydrolyze 7-*O*-glucoside of the icariin or epimedin A, B, or C to icariside II or sagittatoside A, B, or C, respectively. Moreover, it can further hydrolyze terminal 3-*O*-xyloside of the sagittatoside B to icariside II. Thus, the enzyme is a special icariin glycosidase hydrolyzing icariin to icariside II.

Using purified enzyme was of high cost, so the crude enzyme from the *Aspergillus* sp.y848 strain was used to prepare icariside II from icariin isolated from a powdered extract of *Epimedium*. The crude enzyme from *Aspergillus* sp.y48 strain contains a trace of the enzyme hydrolyzing icariside II to icaritin, so the icariside II product contains a small amount of icaritin, but does not have any effect on icariside II production from icariin.

Therefore, using the non-GMO crude enzyme from *Aspergillus* sp.y48 strain, the icariside II was resoundingly produced from icariin separated from the *Epimedium* extract powder using relatively simple and cost-effective methods, and the additional by-product icaritin was also obtained. These results should prove useful in drug development with *Epimedium*.

## Supplemental Materials

Supplementary data for this paper are available on-line only at http://jmb.or.kr.
