# Supplementary material for: Icariside II Preparation from Icariin Separated from Epimedium Herbal Extract Powder by a Special Icariin Glycosidase
Source: J Microbiol Biotechnol. 2024 Oct 24;34(12):2683–92. doi: 10.4014/jmb.2408.08046 (PMC11729362; doi:10.4014/jmb.2408.08046)
Supplement: Supplementary file 1 [file jmb-34-12-2683-supple.pdf]

Supplementary Files

Title: Icariside II preparation from icariin separating from *Epimediun* herb extract-powder by a special icariin-glycosidase

Table S1.

Effects of different substrates on the activity of the purified enzyme

*p*NP Series substrates	Enzyme activity （U/mg）	hydrolysis ability
p-Nitrophenyl-β-L-arabinopyranoside	0.00	-
p-Nitrophenyl-α-D-mannopyranoside	0.00	-
p-Nitrophenyl-α-L-rhamnopyranoside	0.00	-
p-Nitrophenyl-α-D-glucopyranoside	0.07	-
p-Nitrophenyl-β-D-glucopyranoside	3.64	+++
p-Nitrophenyl-β-D-galactopyranoside	0.72	+
p-Nitrophenyl-α-L-fucopyranoside	0.00	-
p-Nitrophenyl-β-D-mannopyranoside	0.00	-
p-Nitrophenyl-α-D-galactopyranoside	0.04	-
p-Nitrophenyl-α-L-arabinopyranoside	0.06	-
p-Nitrophenyl-β-D-fucopyranoside	0.00	-
p-Nitrophenyl-β-D-cellobioside	1.98	++
p-Nitrophenyl-α-D-xylopyranoside	0.00	-
p-Nitrophenyl-β-D-xylopyranoside	0.03	-

Note : + represents that the enzyme has the ability to hydrolyze the substrate, the more the number, the stronger the hydrolysis ability ; - represents that the enzyme has no hydrolysis ability to the substrate.

Table S2.

The enzyme extraction and purification from the culture of *Aspergillus sp.*y48 strain.

Steps	Volume (ml)	Total activity (U)	Total protein (mg)	Specific activity (U/mg)	Purification fold (× fold)	Yield (%)
Enzyme culture	100	4630	368	12.6	1.0	100
Methanol step	10	3012	188	16.0	1.27	65
DEAE-cellulose	15	265	1.56	169	13.4	5.7

Table S3.

Effect of metallic ions on purified special icariin-glycosidase activity from *Aspergillus sp.*y48 strain (Relative activity %).

Concentration（mM/L）	Na^+	K^+	Mg^{2+}	Ca^{2+}	Zn^{2+}	Fe^{3+}	Cu^{2+}
0	100	100	100	100	100	100	100
5	107	99.6	105	106	109	18.1	20.1
10	98.5	104	116	109	70,1	4.05	8.0
50	105	99.8	115	104	60.0	0	0
100	98.0	101	109	102	25.0	0	0
200	98	102	110	103	7.0	0	0

Fig. S1. Structures of main *Epimedium* flavonoids.

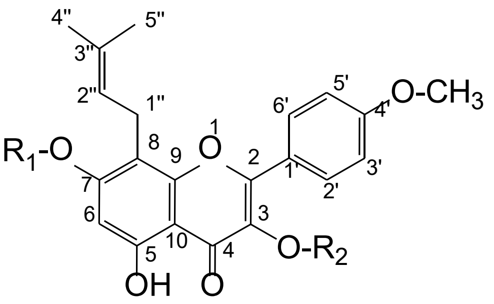

Flavonoids	R_1	R_2	M.W.
**High content**			
Epimedin A	7-*O*-β-D-Glc-	3-*O*-β-D-Glc-(1→2)-α-L-Rha-	822.8
Epimedin B	7-*O*-β-D-Glc-	3-*O*-β-D-Xyl-(1→2)-α-L-Rha-	808.8
Epimedim C	7-*O*-β-D-Glc-	3-*O*-α-L-Rha-(1→2)-α-L-Rha-	822.8
Icariin	7-*O*-β-D-Glc-	3-*O*-α-L-Rha-	676.65
**Rare**			
Sagittatoside A	H	3-*O*-β-D-Glc-(1→2)-α-L-Rha-	676.66
Sagittatoside B	H	3-*O*-β-D-Xyl-(1→2)-α-L-Rha-	646.64
Sagittatoside C	H	3-*O*-α-L-Rha(1→2)-α-L-Rha-	718.71
Icariside I	7-*O*-β-D-Glc-	H	530.52
Icariside II	H	3-*O*-α-L-Rha-	514.5
Icaritin	H	H	368.38

Fig.S1. Structures of main *Epimedium* flavonoids.

Glc, glucoside; Rha, rhamoside; Xyl, xyloside

Fig.S2. Purified enzyme mixture of fractions 78 to 83 from *Aspergillus sp.*y48 in SDS-PAGE and protein HPLC.

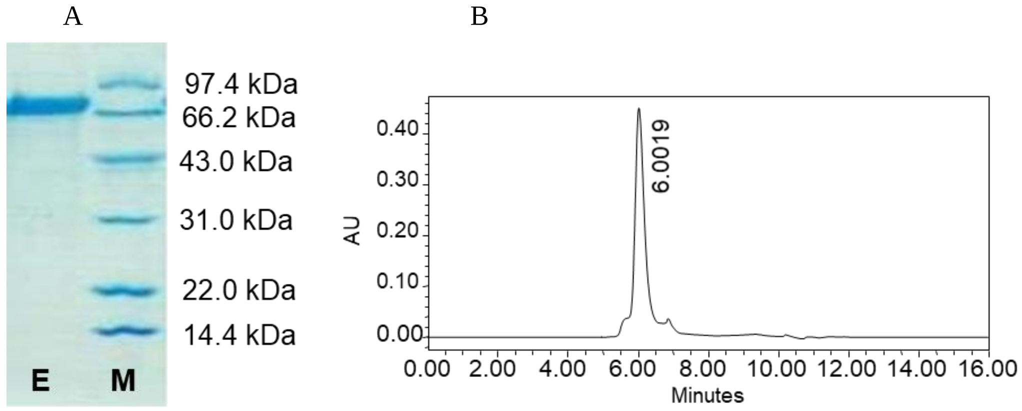

Fig.S2. Purified enzyme mixture of fractions from 79 to 83 from *Aspergillus sp.*y48 in SDS-PAGE and protein HPLC

(A) E, purified enzyme mixture of fractions from 79 to 83; M, marker protein: phosphorylase b (97.4 kDa) ,serum albumin (66.2 kDa), ovalbumin (44.0 kDa), carbonic anhydrase (31 kDa), trypsin inhibitor (20. 2kDa), and lysozyme (14.4 kDa). E, purified enzyme. (B)
purified enzyme mixture of the fractions from 79to 82 in protein HPLC.

In order carefully, the enzyme purity was further examined. The enzyme band of the PAGE gel was cut

out, and dissolved in 0.01 M (pH 5.0) acetate buffer. After removing the non-dissolved material by centrifugation, the resultant enzyme protein was also one band in SDS-PAGE and one peak in protein HPLC. The specific enzyme activity of further purified enzyme was almost same as that of the enzyme purifying by the step of DEAE-Cellulose DE-52 column.

Basing on the protein mobility and plotting the log of the marker protein molecular weight in SDS-PAGE [31,32], the enzyme molecular weight for the enzyme of fractions 78 to 83 was approximately 75 kDa.

Fig. S3. The relationship between the enzyme reaction rate and the substrate concentration obtained by the Lineweaver Burk method

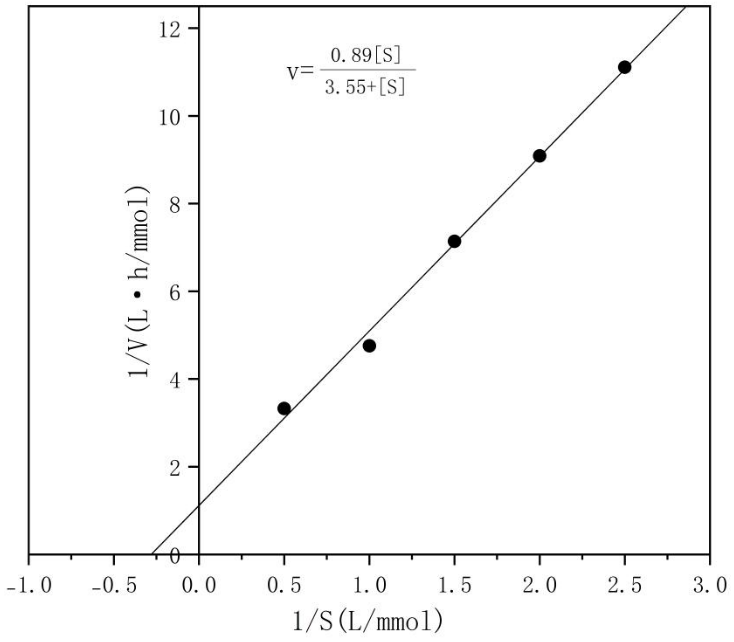

Fig. S3. Double reciprocal diagram of the purified enzyme. V represent the reaction rate, and S represent the substrate concentration.

Fig. S4. Additional information of spectra for enzymatic main producticariside II analyzing by NMR

The icariside II product was dissolved in DMSO-d_6 or Pyridine-d_5, and the NMR spectra were recorded by using the Bruke Avance 600 NMR spectrometer (^{1}H: 600 MHz; ^{13}C: 150 MHz) (Switzerland).

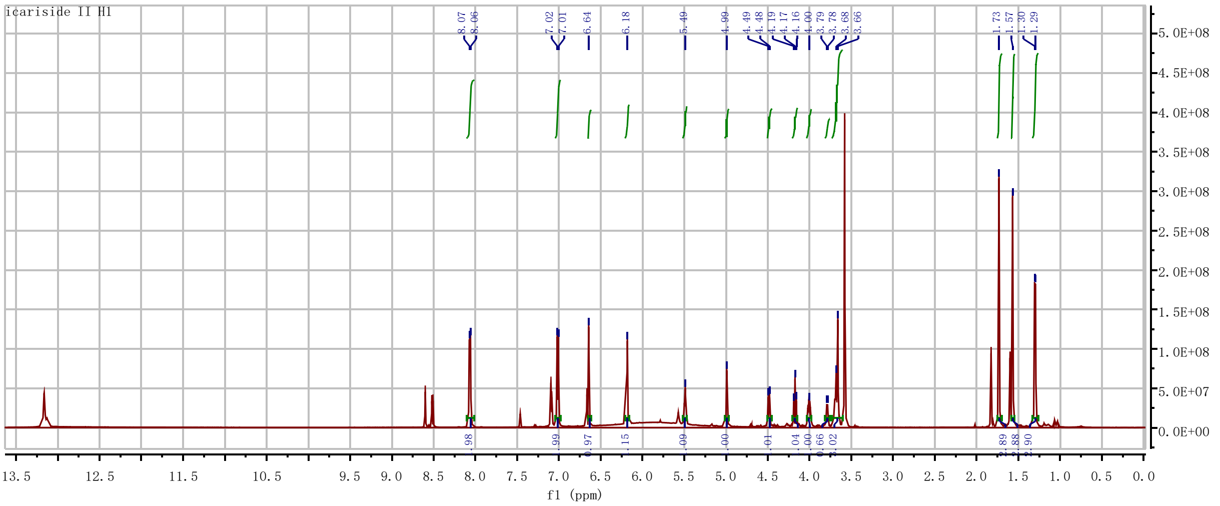

Fig. S4-1. ^{1}H NMR (^{1}H: 600 MHz) of product icariside II

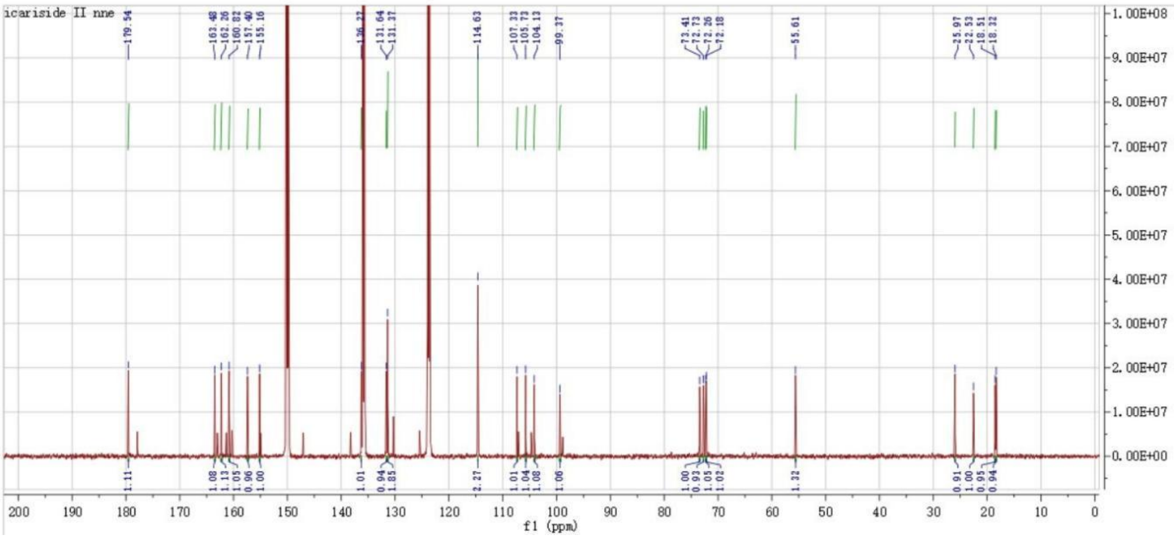

Fig. S4-2. ^{13}C NMR (^{13}C: 150 MHz) of product icariside II

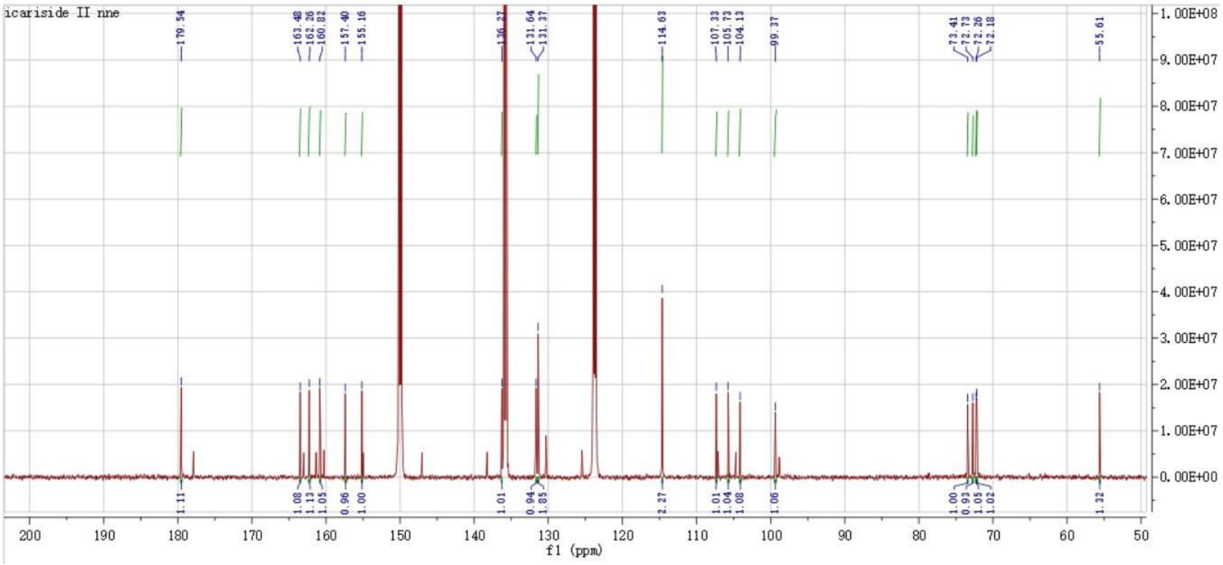

Fig. S4-3. δ50~200 ppm of ^{13}C NMR (^{13}C: 150 MHz) of product icariside II

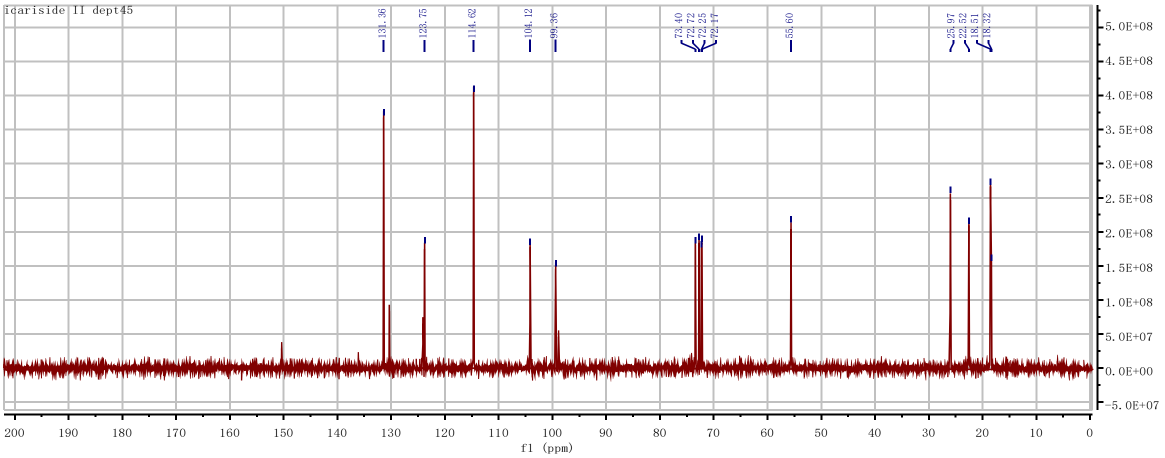

Fig. S4-4. ^{13}C DEPT 45 of product icariside II

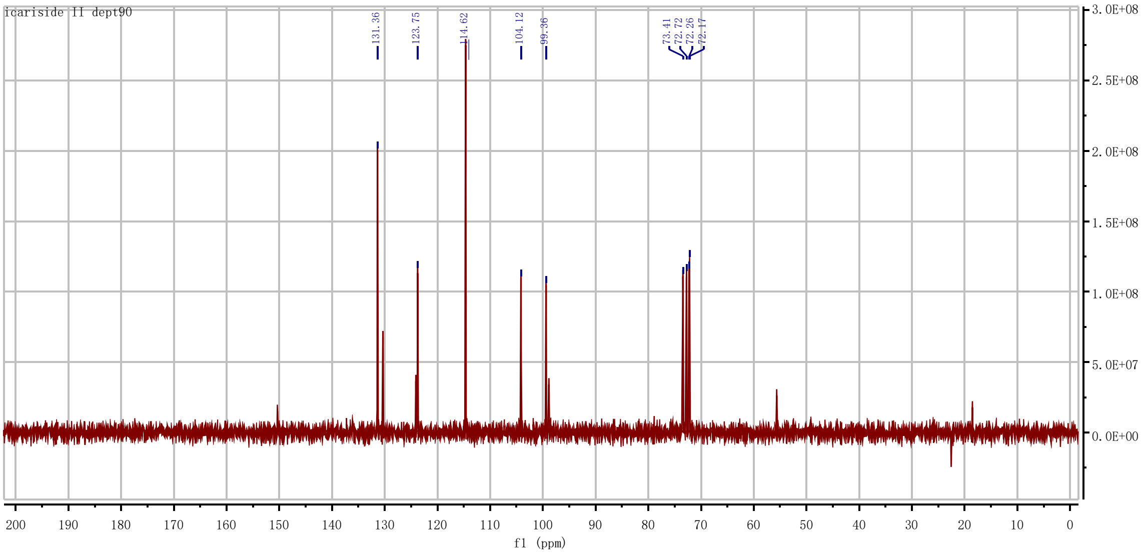

Fig. S4-5. ^{13}C DEPT 90 of product icariside II

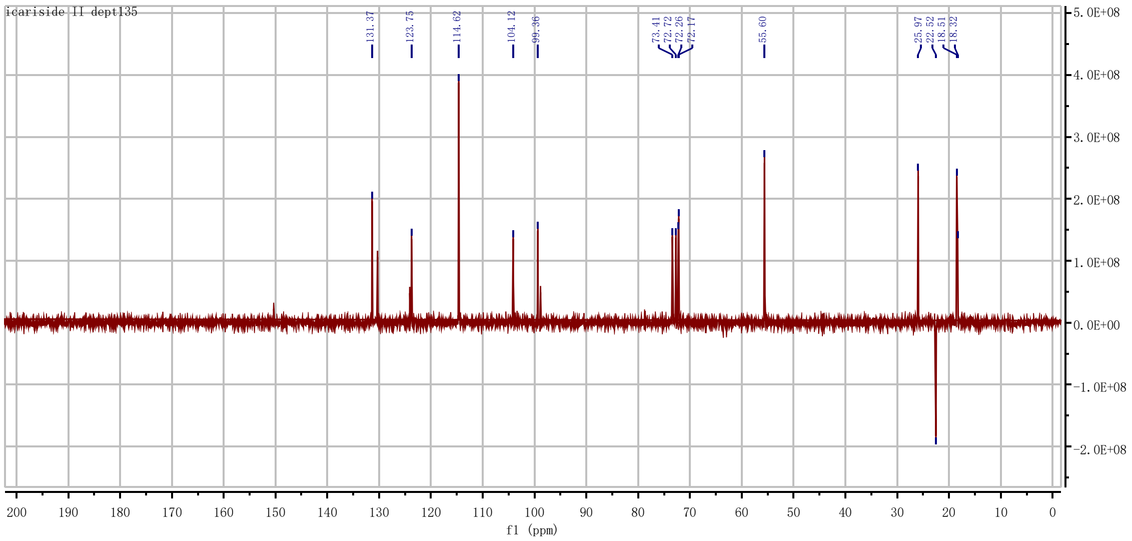

Fig. S4-6. ^{13}C DEPT 135 of product icariside II

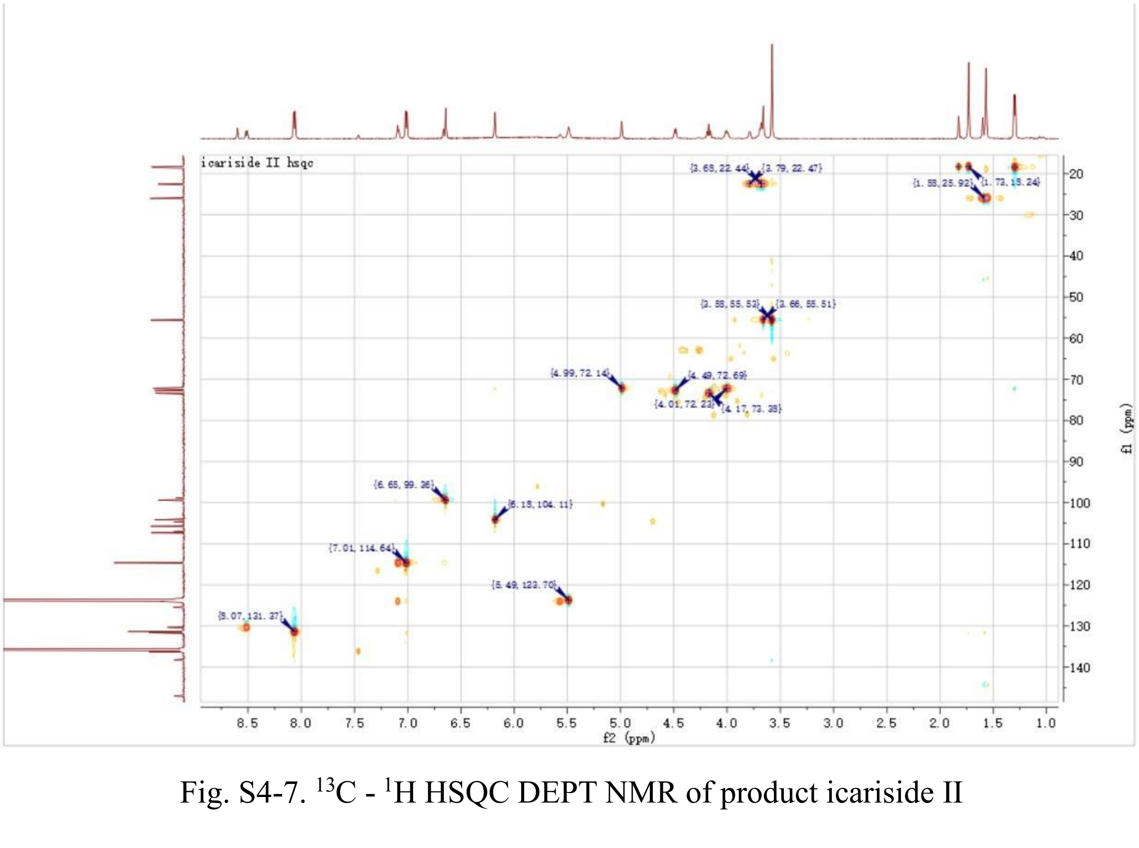

Fig. S4-7. ^{13}C - ^{1}H HSQC DEPT NMR of product icariside II

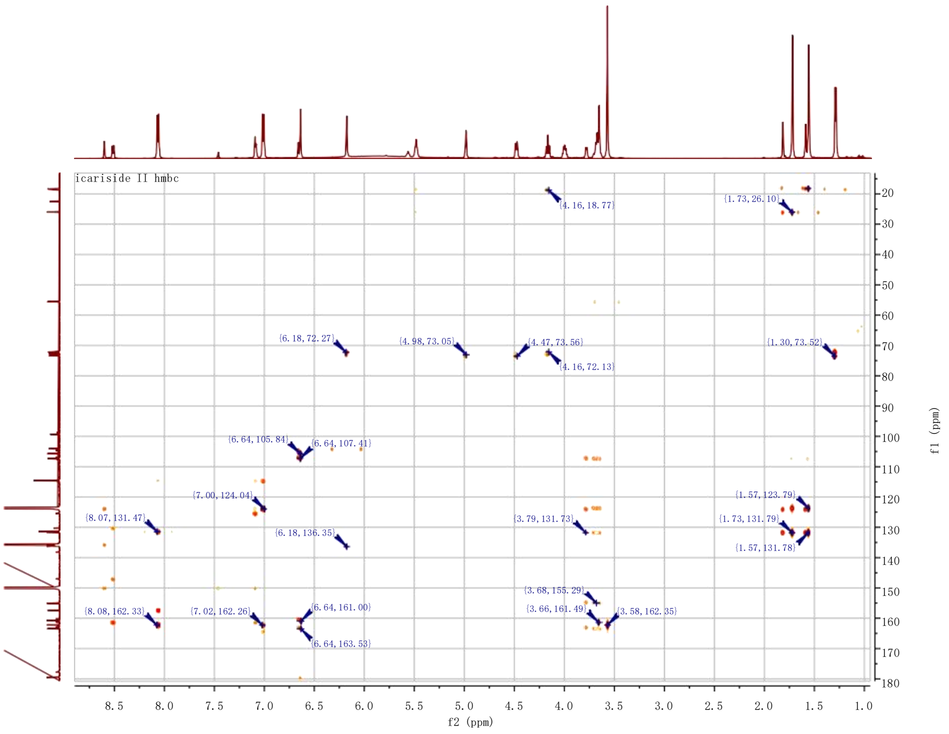

Fig. S4-8. ^{13}C - ^{1}H HMBC DEPT NMR of product icariside II

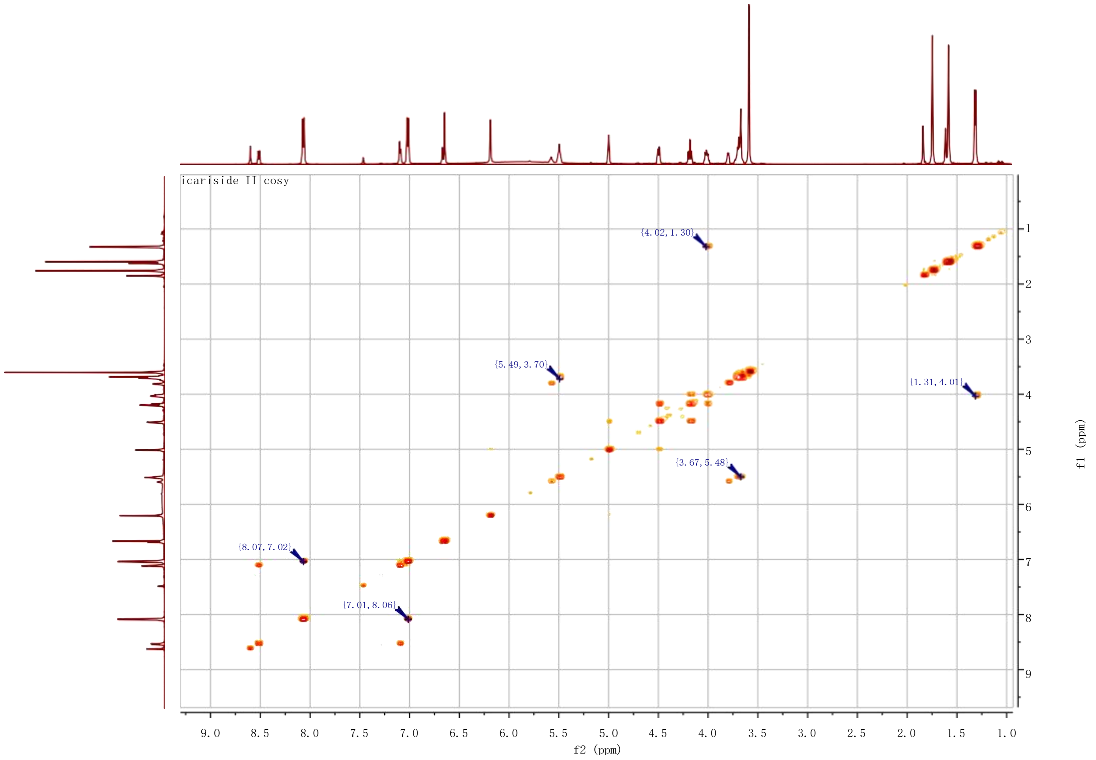

Fig. S4-9. ^{1}H-^{1}H DEPT NMR (^{1}H: 600 MHz) of product icariside II

Fig. S5. Additional information of spectra for enzymatic by-product-icaritinanalyzing by NMR

The structure of enzymatic product icaritin from icariin was analyzed using NMR. The product was dissolved in Pyridine-d_5, and the NMR spectra were recorded by using the Bruke Avance 600 NMR spectrometer (^{1}H: 600 MHz; ^{13}C: 150 MHz) (Switzerland).

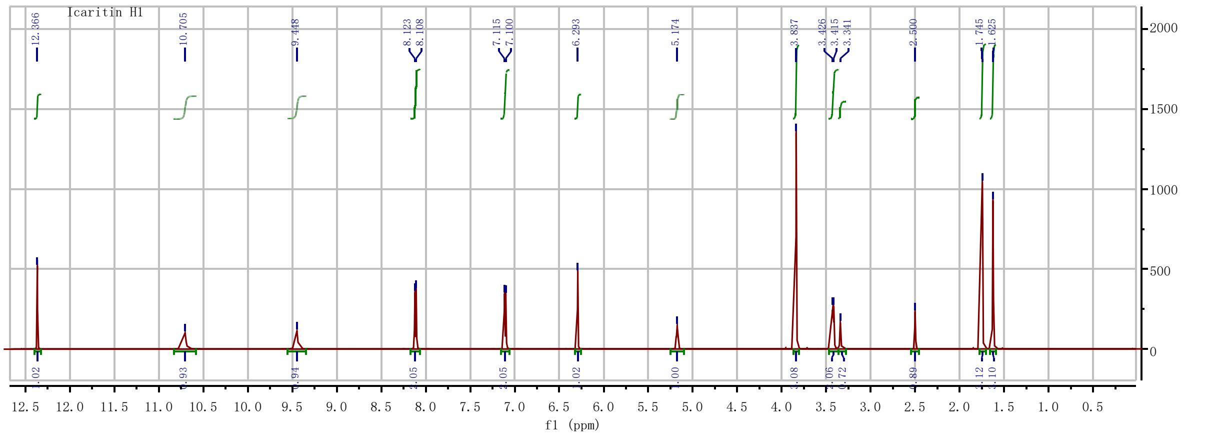

Fig. S5-1. ^{1}H NMR (600 MHz, Pyridine-D$_5$) spectrum of icaritin

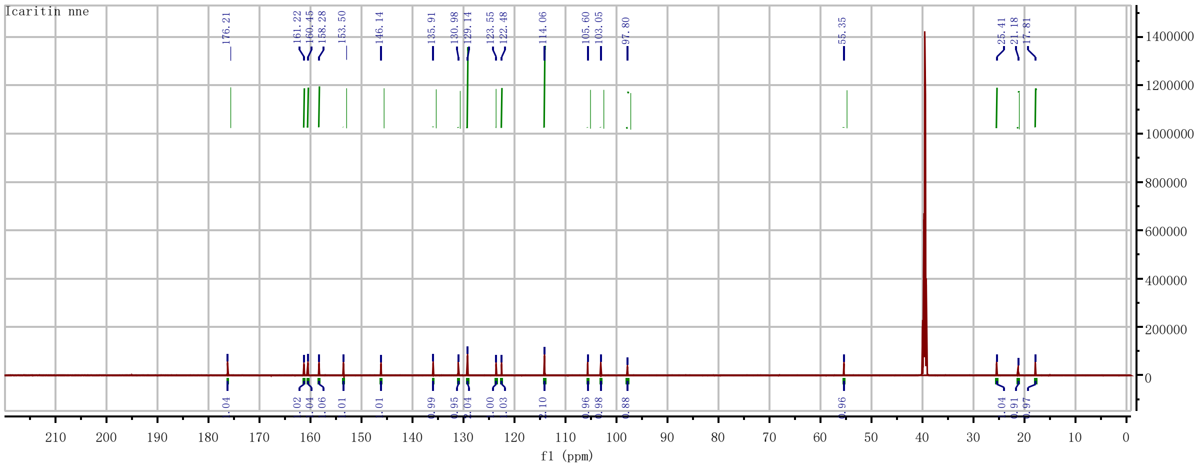

Fig. S5-2. ^{13}C NMR (150 MHz, Pyridine-D$_5$) spectrum of icaritin

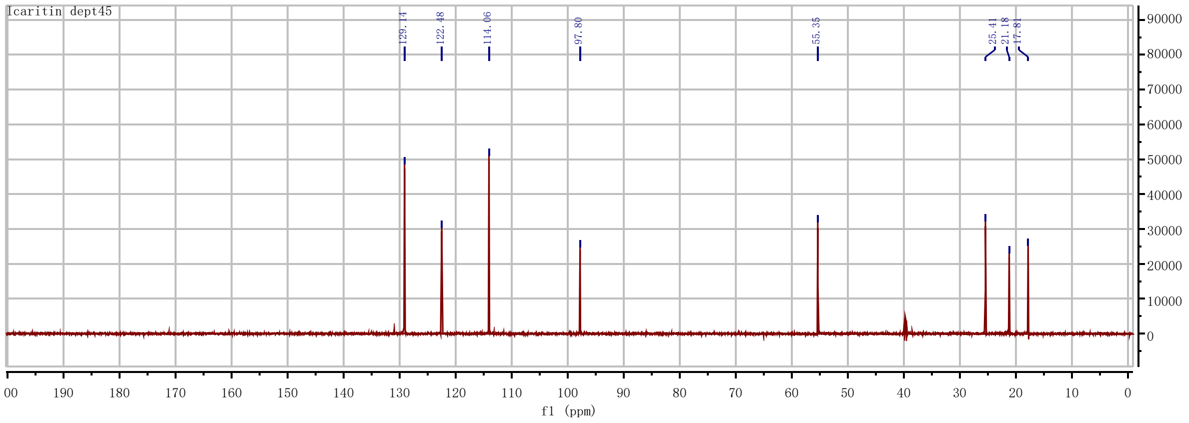

Fig. S5-3. ^{1}H-^{1}H NMR (^{1}H: 600 MHz)C DEPT 45 NMR spectrum of icaritin

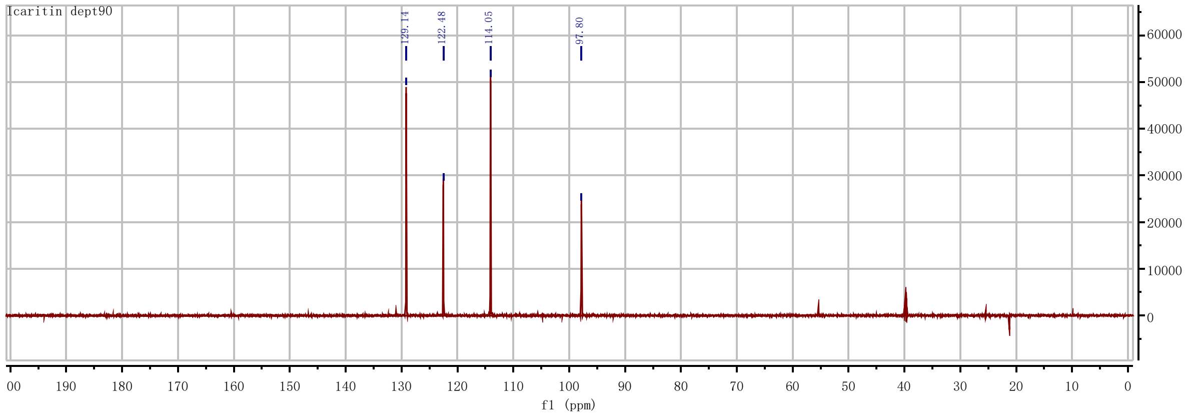

Fig. S5-4. ^{13}C DEPT 90 NMR spectrum of iacritin

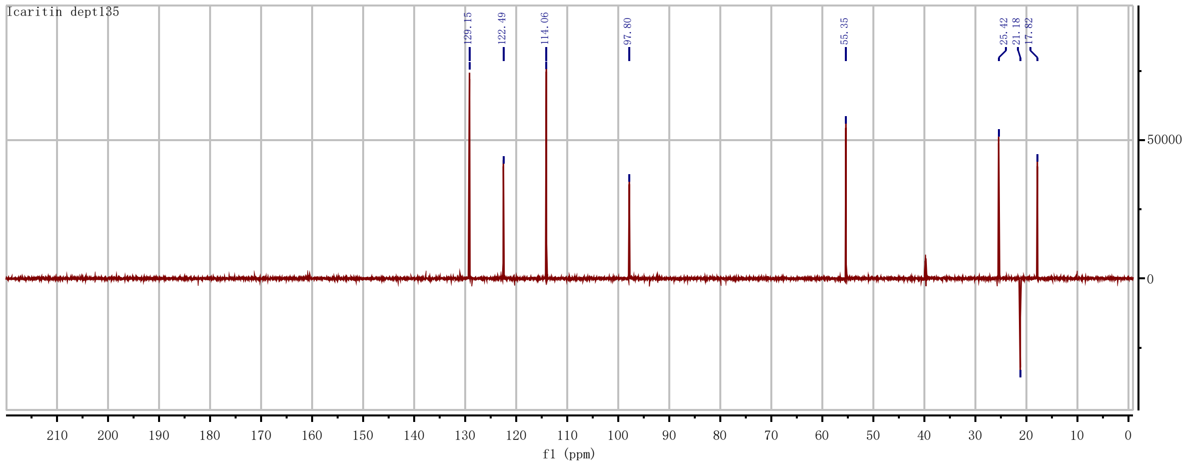

Fig. S5-5. ^{13}C DEPT 135 NMR spectrum of icaritin

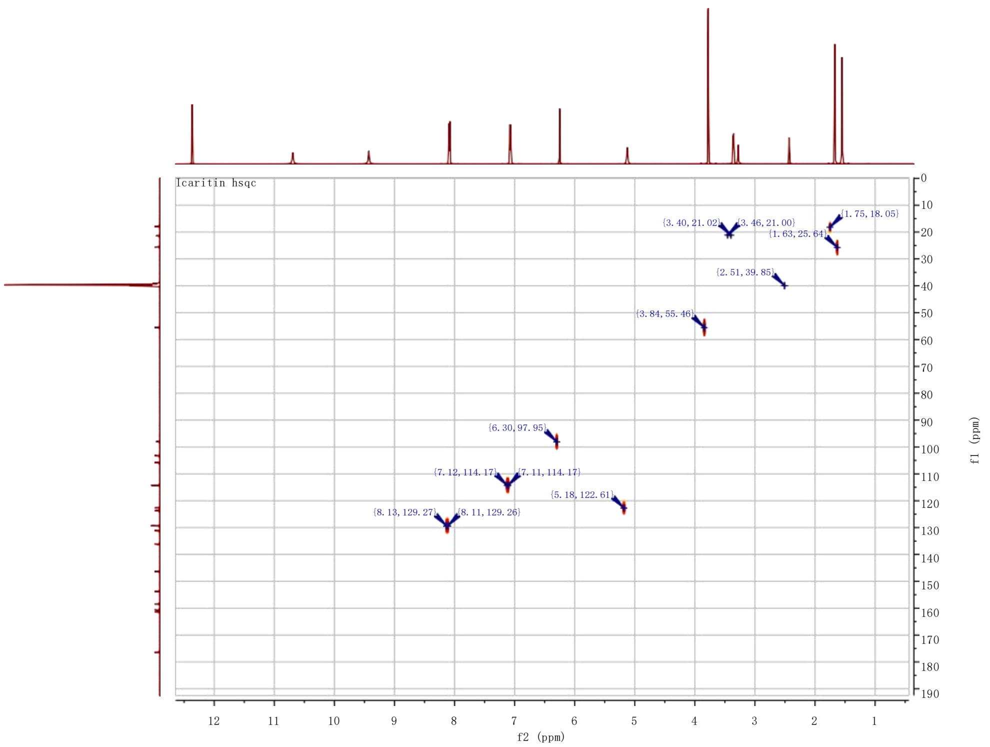

Fig. S5-6. ^{1}H-^{13}C HSQC spectrum of icaritin

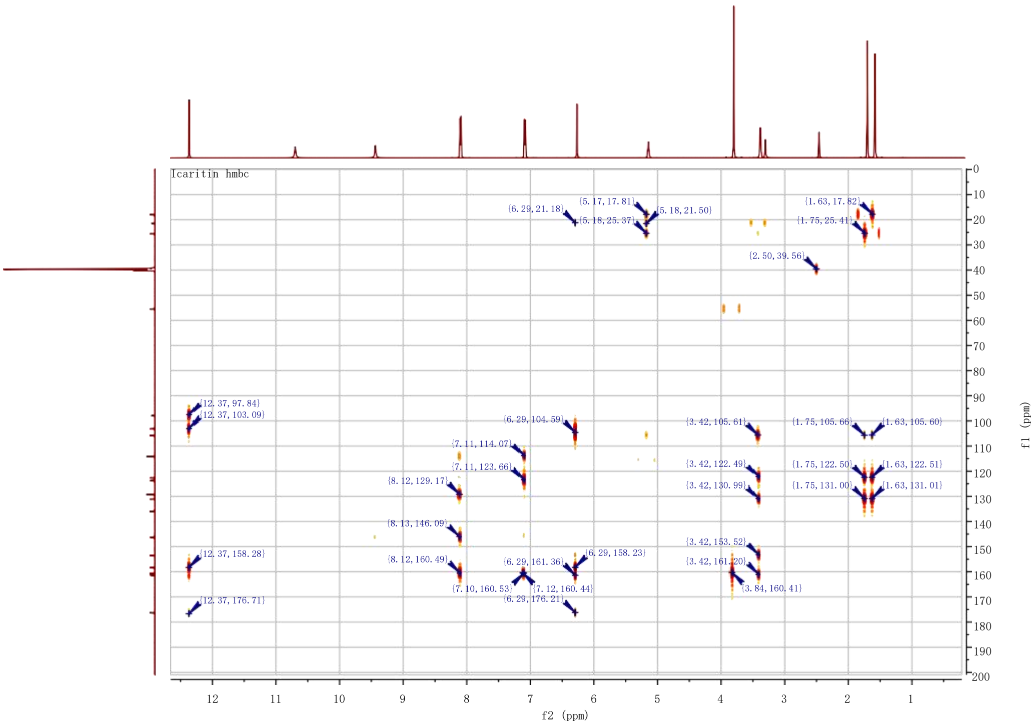

Fig. S5-7. ^{1}H-^{13}C HMBC spectrum of icaritin

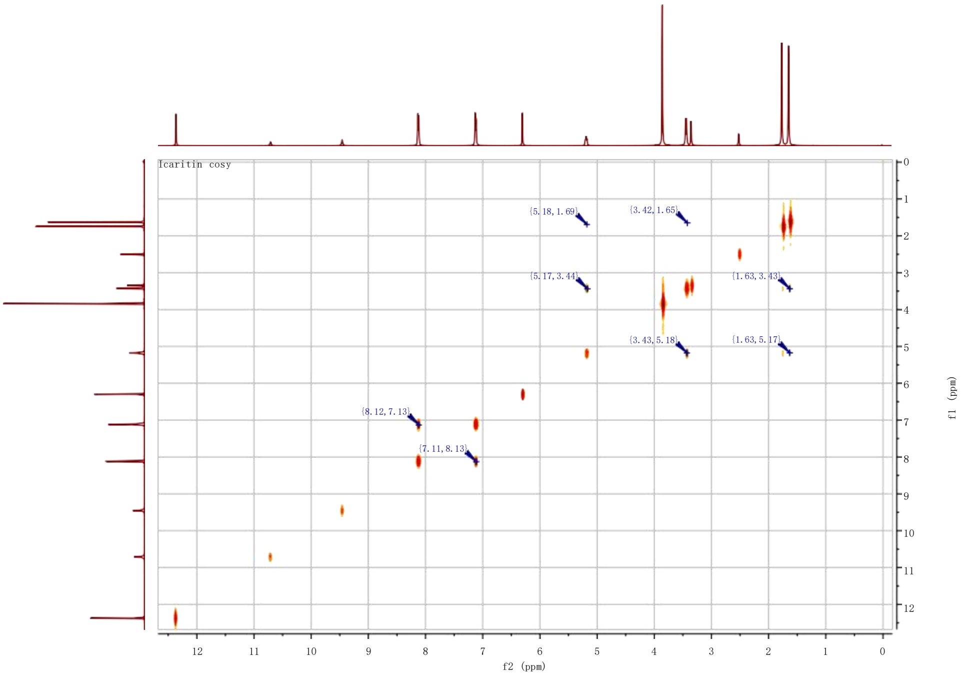

Fig. S5-8. ^{1}H-^{1}H COSY spectrum of icaritin
